# Gait-Phase Modulates Alpha and Beta Oscillations in the Pedunculopontine Nucleus

**DOI:** 10.1523/JNEUROSCI.0770-21.2021

**Published:** 2021-10-06

**Authors:** Shenghong He, Alceste Deli, Petra Fischer, Christoph Wiest, Yongzhi Huang, Sean Martin, Saed Khawaldeh, Tipu Z. Aziz, Alexander L. Green, Peter Brown, Huiling Tan

**Affiliations:** ^1^MRC Brain Network Dynamics Unit, University of Oxford, Oxford OX1 3TH, United Kingdom; ^2^Nuffield Department of Clinical Neurosciences, University of Oxford, Oxford OX3 9DU, United Kingdom; ^3^Nuffield Department of Surgical Sciences, University of Oxford, Oxford OX3 9DU, United Kingdom; ^4^Academy of Medical Engineering and Translational Medicine, Tianjin University, Tianjin 300072, People's Republic of China; ^5^Oxford Centre for Human Brain Activity, Wellcome Centre for Integrative Neuroimaging, University of Oxford, Oxford OX3 7JX, United Kingdom

**Keywords:** deep brain stimulation, freezing of gait, gait phase-related modulation, multiple system atrophy, Parkinson's disease, pedunculopontine nucleus

## Abstract

The pedunculopontine nucleus (PPN) is a reticular collection of neurons at the junction of the midbrain and pons, playing an important role in modulating posture and locomotion. Deep brain stimulation of the PPN has been proposed as an emerging treatment for patients with Parkinson's disease (PD) or multiple system atrophy (MSA) who have gait-related atypical parkinsonian syndromes. In this study, we investigated PPN activities during gait to better understand its functional role in locomotion. Specifically, we investigated whether PPN activity is rhythmically modulated by gait cycles during locomotion. PPN local field potential (LFP) activities were recorded from PD or MSA patients with gait difficulties during stepping in place or free walking. Simultaneous measurements from force plates or accelerometers were used to determine the phase within each gait cycle at each time point. Our results showed that activities in the alpha and beta frequency bands in the PPN LFPs were rhythmically modulated by the gait phase within gait cycles, with a higher modulation index when the stepping rhythm was more regular. Meanwhile, the PPN–cortical coherence was most prominent in the alpha band. Both gait phase-related modulation in the alpha/beta power and the PPN–cortical coherence in the alpha frequency band were spatially specific to the PPN and did not extend to surrounding regions. These results suggest that alternating PPN modulation may support gait control. Whether enhancing alternating PPN modulation by stimulating in an alternating fashion could positively affect gait control remains to be tested.

**SIGNIFICANCE STATEMENT** The therapeutic efficacy of pedunculopontine nucleus (PPN) deep brain stimulation (DBS) and the extent to which it can improve quality of life are still inconclusive. Understanding how PPN activity is modulated by stepping or walking may offer insight into how to improve the efficacy of PPN DBS in ameliorating gait difficulties. Our study shows that PPN alpha and beta activity was modulated by the gait phase, and that this was most pronounced when the stepping rhythm was regular. It remains to be tested whether enhancing alternating PPN modulation by stimulating in an alternating fashion could positively affect gait control.

## Introduction

The pedunculopontine nucleus (PPN) is a reticular collection of neurons at the junction of midbrain and pons (Jenkinson et al., 2009; Thevathasan and Moro, 2019). Its main mass is part of the mesencephalic locomotor region (MLR) in the upper brainstem, and it is thought to play an important role in modulating posture, locomotion, and arousal (Saper et al., 2001; Tattersall et al., 2014; Sébille et al., 2017). Electrical stimulation of the MLR that directly projects to the locomotor central pattern generators in the spinal cord can generate walking, trotting, and galloping in a decerebrated cat (Shik et al., 1966), and can transition between swimming-like and walking-like movements in the salamander (Cabelguen et al., 2003). Deep brain stimulation (DBS) of the PPN has been proposed as an emerging treatment for patients with Parkinson's disease (PD) with gait problem (Ferraye et al., 2010; Moro et al., 2010), and for patients with multiple system atrophy (MSA) who have gait-related atypical parkinsonian syndromes (Ostrem et al., 2010; Acar et al., 2011; Rowe et al., 2016). However, the therapeutic efficacy of PPN DBS and the extent to which it can improve quality of life seem too variable to draw firm conclusions, rendering it still an experimental therapy (Thevathasan et al., 2018). This could be because of the still unclear complex structure–function relationships between these structures and locomotion (Fasano et al., 2015; Pienaar et al., 2017).

Conventional DBS settings deliver electrical pulses continually at a fixed frequency to the target brain area. Considering the rhythmic structure of movements during gait, conventional continuous DBS may be suboptimal for improving gait control (Fischer et al., 2020). Understanding how neural activity in the PPN is modulated during gait could be a crucial step in improving the therapeutic effects of PPN DBS. Increased alpha power in the PPN has been observed in human patients during locomotion, including free walking or imaginary gait (Thevathasan et al., 2012; Tattersall et al., 2014; Lau et al., 2015), and increased alpha power in the PPN during walking correlated with gait performance (Thevathasan et al., 2012). Similarly, increased low-frequency oscillations in the PPN in the 1–8 Hz range have also been reported (Molina et al., 2020). However, the studies reported a negative relationship between increased power in this low-frequency band and movement speed especially when patients were off medication, which conflicts with some of the previous literature. More recently, Molina et al. (2021) reported a low-frequency (1–8 Hz) power-triggered closed loop PPN DBS study and showed a 40% improvement in medication-refractory freezing of gait (FoG) in three of five PD patients with FoG. However, there is no comparison of the low-frequency power triggered closed loop PPN DBS against continuous PPN DBS, so it is still difficult to establish the relationship between the low-frequency oscillations in the PPN and gait.

The previous literature so far has focused on the changes in the average power of the oscillatory activities in different frequency bands during walking compared with resting or standing still. Whether and how the activities in PPN LFPs are modulated within a gait cycle during walking or stepping are still unknown. This study aims to address this question as this may lead to new patterns of PPN stimulation that are more effective in ameliorating gait difficulties and facilitate rhythmic walking. To this end, we recorded PPN LFPs from patients undergoing DBS surgery targeting PPN for gait difficulties during stepping or free walking with simultaneous measurements from force plates or an accelerometer attached to the trunk to determine the phase within each gait cycle of each time point. Our results show that PPN activities in the alpha and beta frequency bands are rhythmically modulated by the phase of gait. The modulation is higher during more regular stepping, and the modulation is spatially limited to the PPN area.

## Materials and Methods

### 

#### Ethics

The present study was conducted in accordance with the Declaration of Helsinki, approved by the local ethics committees of the University of Oxford Hospitals or University College London Hospitals NHS foundation trust. All patients provided informed written consent before the experiment.

#### Participants

Data were recorded from seven PD patients and four MSA patients who underwent bilateral (*n* = 9) or unilateral (*n* = 2) implantation of DBS electrodes targeting the PPN. The average (±SD) age and disease duration of these patients (all male) were 65.45 ± 8.51 and 11.95 ± 8.53 years, respectively. The DBS electrodes were implanted and temporarily externalized (3–7 d) before a second surgery to connect the leads to a pulse generator in all patients. Six PD patients received the Medtronic electrode model 3389 (0.5 mm spacing between contacts) and one PD patient received the Medtronic electrode model 3387 (1.5 mm spacing between contacts). All MSA patients were implanted with 1.5 mm spaced St. Jude Medical Infinity directional DBS electrodes (Abbott). The placements of the electrodes were confirmed by the fusion of preoperative MRI and postoperative CT scans. All experiments were conducted after overnight withdrawal of all dopaminergic medication. In total, data recorded from 20 PPNs were analyzed in this study [the data pertaining to average power changes during walking from all the seven PD patients (12 hemispheres) have been previously reported; Thevathasan et al., 2012]. The clinical details of the patients are summarized in Table 1.

**Table 1.** Clinical details of all patients

Patients	G, age (years)	DD (years)	DBS lead	UPDRS- III OFF/ON medication (score/108)	IT27/30 OFF/ON medication (score/16)	UMSARS-II	l-DOPA dose equivalent (mg/d)	Other preoperative medication	Data and task
PD01	M, 71	20	Met1	37/19	10/5	NA	1450	NA	Rest sitting; rest standing; free walking
PD02	M, 55	25	Met2	33/22	6/5	NA	300	NA	Rest sitting; rest standing; free walking
PD03	M, 68	9	Met2	40/26	11/8	NA	1650	NA	Rest sitting; rest standing
PD04	M, 55	14	Met2	35/24	7/6	NA	1600	NA	Rest sitting; rest standing
PD05	M, 70	20	Met2	35/22	6/5	NA	900	NA	Rest sitting; rest standing
PD06	M, 76	16	Met2	34/25	9/9	NA	600	NA	Rest sitting; rest standing
PD07	M, 54	20	Met2	53/19	6/5	NA	800	NA	Rest sitting; rest standing
MSA01	M, 74	2	Abb	NA	NA	16	NA	Ropinirole: 14 mg once/d	Rest sitting; step sitting; step standing
MSA02	M, 72	2	Abb	NA	NA	12	NA	Co-beneldopa: 50/12.5 mg, two capsules 3 times/d	Rest sitting; step sitting; step standing
MSA03	M, 71	2	Abb	NA	NA	17	NA	Co-careldopa: 25 mg/100 mg tablets; dose, one tablet 3 times/d	Rest sitting; step sitting
MSA04	M, 54	1.5	Abb	NA	NA	27	NA	Co-beneldopa: 25 mg/100 mg tablets; dose, two capsules 4 times/d Rasagiline: 1 mg once/d Amantadine: 100 mg twice/d	Rest sitting; step sitting
Mean	65.45	11.95	NA	38.14/22.43	7.86/6.14	18	1042.86	NA	NA
SD	8.51	8.53	NA	6.42/2.56	1.96/1.55	5.52	488.75	NA	NA

All patients were operated on in Oxford except PD01 (operated on in London). For all motor scales, higher scores indicate worse function. The MSA patients had been attributed to Parkinson's disease before clinically diagnosis of MSA. G, Gender; M, male; DD, disease duration; UPDRS-III, motor subsection (part III) of unified Parkinson's disease rating scale; IT27/30, items 27–30 of UPDRS-III assessing posture, gait, and balance; Abb, infinity 1.5 mm spaced leads (1–4), Abbott; Met1, model 3387 1.5 mm spaced leads, Medtronic; Met2, model 3389 0.5 mm spaced leads, Medtronic; NA, not available.

#### Experimental protocol

A rest recording during which patients sat comfortably and relaxed with eyes open for 2–3 min was completed for each patient. Each PD patient completed another rest recording while standing comfortably for 2–3 min followed by a gait recording, where patients walked at their preferred speed along an unobstructed path (∼10 m) for 10–30 times, depending on speed and fatigue. Each MSA patient completed a stepping-in-place recording during which they stepped on two pressure sensor plates (Biometrics) guided by a walking cartoon man displayed on a laptop while sitting comfortably in a chair, with a metronome sound provided at the time of each heel strike of the cartoon man, similar to the paradigm used in the previous studies (Fischer et al., 2018; Tan et al., 2018). Two MSA patients (MSA01, MSA02) with less severe gait problems were also recorded while stepping on the spot while standing. The participants were asked to follow the stepping rhythm of the walking cartoon man as precisely as possible, with one complete cycle (i.e., one right step and one left step) lasting 2 s. Because of the severity of the symptoms and the risk of falling, we were not able to perform the free walking task on MSA patients.

#### Recordings

For directional DBS leads, all segmented contacts of level 2 or 3 were physically jointed together to make one monopolar contact, resulting in four monopolar contacts from each DBS electrode. PPN LFPs and EEGs covering “Fz,” “F3,” “F4,” “Cz,” “C3,” “C4,” “Pz,” “Oz,” “CP3,” and “CP4” according to the standard 10–20 system (with some variability in terms of EEG electrode location depending on the accessibility after DBS surgery) were also recorded for all patients except one (no EEGs for PD07), in a monopolar configuration with a common reference rejection. In six PD patients (PD01 to PD06), triaxial accelerometers were taped over the upper thoracic spinous processes to record their trunk accelerations. In all MSA patients, stepping force from left and right feet were recorded separately using two pressure sensor plates (Biometrics). All of these signals were simultaneously recorded using an amplifier (TMSi Porti, TMS International) with a sampling rate of 2048 Hz. The ground electrode of the amplifier was connected to the chest or the wrist of the patient. A first-order low-pass filter with a −3 dB point at 4.8 kHz and a digital sinc3 filter with a cutoff frequency of 553 Hz were implemented in the amplifier and were applied automatically on all recorded signals. In addition, a common reference rejection was applied automatically on all recorded monopolar EEGs and LFPs by the amplifier.

#### Data analysis

##### Preprocessing.

The recorded data were first visually explored using Spike2 (version 7.02a, Cambridge Electronic Design), and those gait blocks/channels with obvious movement-related artifacts (e.g., big jitter in the time course or increased power over a broad frequency band during gait) were rejected. Then the data were analyzed offline with MATLAB (version 2020a; MathWorks). We first reconstructed spatially focal bipolar LFPs using each pair of two spatially adjacent electrode contacts (e.g., 0–1, 1–2, and 2-3) and bipolar EEGs between Fz and Cz. Compared with monopolar signals, bipolar recordings capture more spatially focal signals and can help to eliminate common activities, such as volume-conducted activity or artifacts (Spaak et al., 2012; Marmor et al., 2017; Nazmuddin et al., 2018). Then, all bipolar LFPs and EEGs were band-stop filtered at 48–52 Hz using a zero-phase eighth-order Butterworth IIR band-stop filter, followed by a zero-phase eighth-order Butterworth IIR bandpass filter at 0.5–250 Hz. The recorded stepping force or accelerometer measurements were bandpass filtered at 0.5–5 Hz using a zero-phase sixth-order Butterworth IIR bandpass filter.

##### Power spectral density and PPN–cortical coherence.

The filtered bipolar LFPs and EEGs were further decomposed into the time–frequency domain by applying continuous complex Morlet wavelet transforms with a linear frequency scale ranging from 1 to 95 Hz and a linearly spaced number (4–8) of cycles across all calculated frequencies. The LFP and EEG power spectral densities (PSDs) were further estimated by averaging the wavelet power across the time periods of interest during resting, stepping, or walking, and then normalized by the sum of the whole frequency band between 1 and 95 Hz, resulting in PSDs in percentage. In addition, based on the time–frequency decomposition results after the complex Morlet transform, the imaginary coherence (IC) between each bipolar LFPs and EEGs (“CzFz”) were calculated according to the following equation (Carter, 1987; Nolte et al., 2004):

(1) $$\text{IC}=\left|\frac{\text{imag}(G_{\text{lfpeeg}})}{\sqrt{G_{\text{lfp}}\text{ * }G_{\text{eeg}}}}\right|,$$

where $G_{\text{lfpeeg}}$, $G_{\text{lfp}}$, and $G_{\text{eeg}}$ indicate the cross-spectral density between LFPs and EEGs, the auto spectral density of LFPs, and EEGs, respectively. The imaginary coherence was computed to avoid spurious increases in coherence that may occur because of artifacts with a time lag of zero.

##### Gait phase determination.

**Figure 1.**
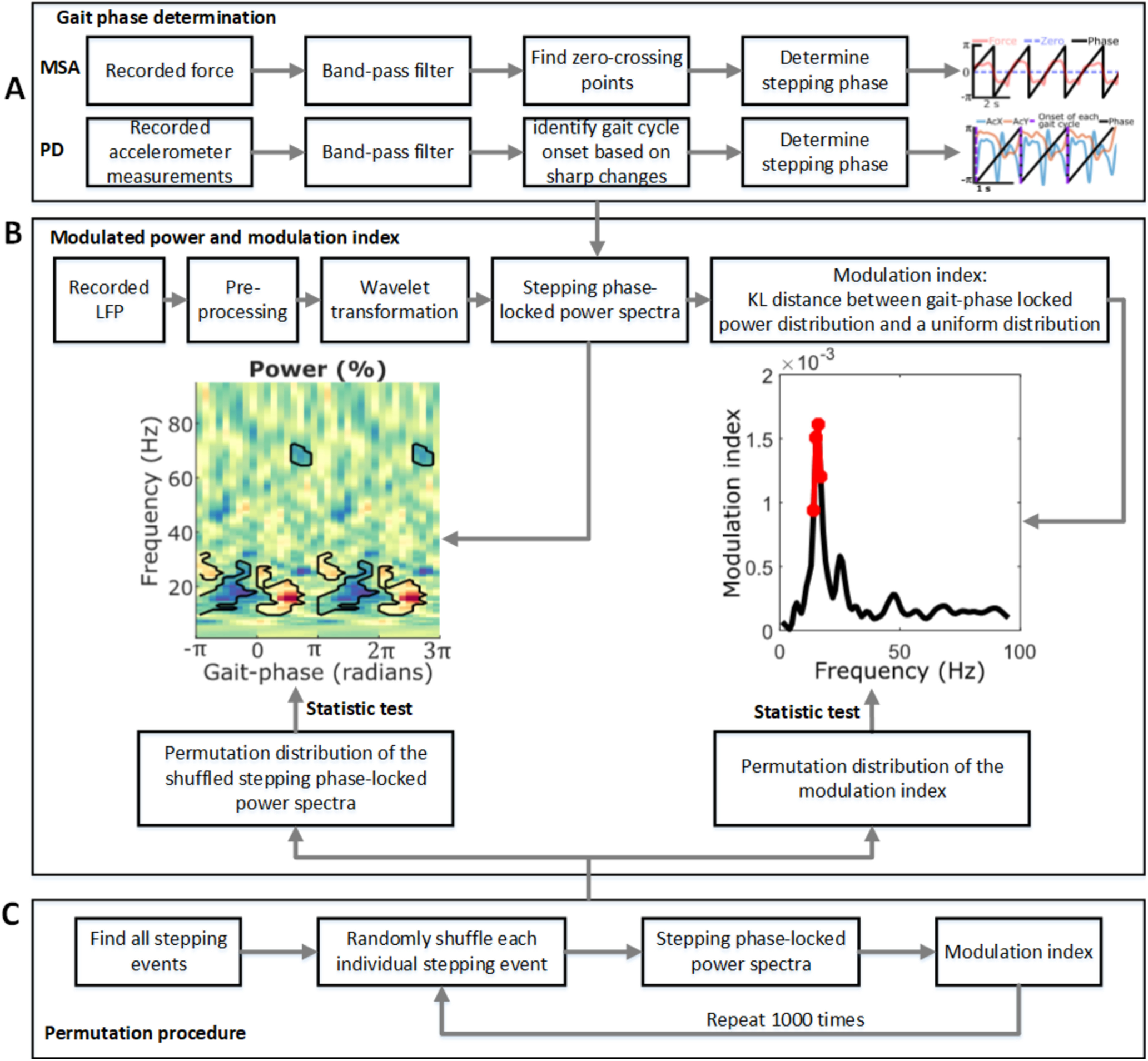
***A–C***, Flowchart for the data analysis procedure, including gait phase determination (***A***), modulated power and modulation index quantification (***B***), and a permutation procedure for statistical analysis (***C***). The black contours and red dots in ***B*** indicate statistical significance according to a cluster-based nonparametric statistical test, which compared the original unpermuted data generated in ***B*** against the permutation distribution generated in ***C*** at a significance level of *p* < 0.05.

To investigate the gait phase-related neural oscillations in the PPN, we determined the phase in a gait cycle (assuming one complete gait cycle to be 2π) for each time point using the recorded force signals or accelerometer measurements. Specifically, when stepping force measurements were available (in the stepping paradigm with all MSA patients), we first find all zero-crossing points from the bandpass-filtered force data. Then, the zero-crossing points where the force decreased, which correspond to the time points when the foot started to lift up, were assigned as phase π or –π; the zero-crossing points where the force increased, which correspond to the time points of foot touching onto the force plate and starting to carry weight, were assigned as phase 0. The phases of all other time points were determined through linear interpolation between –π and 0 or between 0 and π, as shown in Figure 1*A*. In the patients with whom recordings were performed during free walking, we did not have continuous force measurements. In this case, we used measurements from the accelerometers attached to the upper thoracic spinous processes to manually identify each individual gait cycle. However, we managed to identify the gait cycle on only two patients (PD01 and PD02) because there was no clearly distinguishable rhythmic pattern in the accelerometer measurements during walking in the other PD patients. For these two patients, time points with a minimum acceleration value on the *x*-axis and increasing acceleration value on the *y*-axis were determined as phase –π or π. The phases of all other time points were determined through linear interpolation between –π and π, as shown Figure 1*A*. The methods used to determine gait phase using accelerometer measurements were similar to what was used in the study by Fischer et al. (2018). However, the experimental protocol for PD patients was designed specifically for the previous studies by Thevathasan et al. (2011, 2012), and we were only able to detect the gait phase reliably for two patients. Here the phase was equivalent to the percentage of time in a complete stepping/walking cycle, and the time points determined as phase 0 or π based on accelerometer measurements may not match the phase 0 or π determined based on recorded force for the MSA patients.

##### Power modulated by gait phase and the modulation index.

Based on the wavelet power and the identified stepping/walking phase, we estimated LFP activity–gait phase modulogram for each individual bipolar LFPs for the six patients (MSA, four patients; PD 2 patients). Specifically, 18 nonoverlapping bins with equally spaced center phase values were defined from –π to π. Then, the mean power within each phase bin was calculated for each frequency and normalized against the mean of all bins to give a percentage of power for each phase bin (Fig. 1*B*). We further quantified a modulation index (MI) for each frequency. To this end, we first normalized the power in each bin using the sum rather than the mean of all bins to avoid negative values. Then, the MI was defined based on the Kullback–Leibler (KL) distance between the gait phase-locked power at each frequency and a uniform distribution as follows (Kullback and Leibler, 1951; Tort et al., 2010):

(2) $$\text{MI}=\frac{D_{\text{KL}}(P,U)}{\text{log}(N)}$$

(3) $$D_{\text{KL}}(P,U)=\sum_{j=1}^{N}P(j)\text{log}\left[\frac{P(j)}{U(j)}\right],$$

where *P* and *U* indicate the gait phase-locked power distribution at a specific frequency and a uniform distribution. *N* indicates the number of bins (i.e., 18). Here the KL distance is used to infer the amount of difference between two distributions, which has the property that $D_{\text{KL}}\left(P,U\right)\geq 0$ and $D_{\text{KL}}\left(P,U\right)=0$ if and only if when *P* = *U* (i.e., when the distributions are the same). Large deviations from the uniform distribution result in a large modulation index.

##### Clustering of underlying states in PPN LFPs using hidden Markov model and K-medoids.

We also investigated underlying states defined by different dynamics in the PPN LFPs and how the state occupancy changes with gait phase using the HMM-MAR, a method combining the multivariate autoregressive (MAR) model and the hidden Markov model (HMM; Vidaurre et al., 2016). To do so, the filtered bipolar LFPs were first downsampled to 256 Hz. The HMM-MAR modeling was performed on each patient separately, with the LFPs from all bipolar channels from both hemispheres were included in a matrix as the input. The maximum number of states was set to 30. In the HMM-MAR modeling, the states correspond to unique patterns of brain activity that recur in different parts of the time series, and each time point would be assigned to a specific state. Then we quantified the occurrence rate of each state at different gait phase bins, resulting in one histogram for each state. Finally, another unsupervised method, K-medoids (Park and Jun, 2009), was used to cluster these histograms into three main clusters to see how the occupancy of different states changes with the gait phase.

##### Electrode trajectories reconstruction using lead DBS.

To investigate the spatial distribution of the gait phase-related modulation, we further reconstructed the electrode trajectories and location of different contacts using a MATLAB toolbox Lead-DBD (version 2.3.2) based on preoperative magnetic resonance imaging (MRI) and postoperative computed tomography (CT) scans for the five patients with identified gait phase (the scans for PD01 were not available; Horn et al., 2019). The electrode locations were registered and normalized into the Montreal Neurologic Institute (MNI) 152-2009b space using the Harvard Ascending Arousal Network (AAN) Atlas (Edlow et al., 2012). Then, the modulation index and PPN–cortical coherence during rest and gait in different frequency bands (alpha, 8–12 Hz; beta, 13–30 Hz; theta, 4–7 HZ; gamma, 55–95 Hz) for each individual bipolar contact from all five patients were normalized such that the minimum and maximum values were 0 and 1, respectively, in alpha and beta bands and were mapped into the MNI space.

#### Statistical analysis

A nonparametric cluster-based permutation procedure was applied to identify significant gait cycle-related power increases or decreases in time–frequency plots while controlling for multiple comparisons (Maris and Oostenveld, 2007). To compare the group averages between two conditions (rest during sitting vs rest during standing or rest during sitting vs rest during stepping), the condition labels of the original samples were randomly permutated 1000 times such that for each hemisphere the order of subtraction can change. To test the significance of points in the modulogram and the modulation index for individual bipolar contact recordings, we computed a permutation distribution by randomly shuffling the phase for each individual gait cycle. This was done by dividing the original phase vector for each gait cycle into two segments according to a randomly selected point and then swapping back and forth. The randomly selected point to shuffle the phase within each cycle was different for different gait cycles. Based on the shuffled phase vector, we calculated the modulated power and modulation index. This was repeated 1000 times to obtain a permutation distribution with 1000 samples (Fig. 1*C*). This permutation procedure resulted in a null hypothesis distribution of 1000 samples, and the permutation distribution mean and SD were used to *z*-score the original unpermuted data and each permutation sample and obtain a *p* value for each data point. Then, suprathreshold clusters were obtained for the original unpermuted data and each permutation sample by setting a precluster threshold (*p* < 0.05). If the absolute sum of the *z*-scores within the original suprathreshold clusters exceeded the 95th percentile of the 1000 largest absolute sums of *z*-scores from the permutation distribution (i.e., *p* < 0.05), it was considered statistically significant.

## Results

### PPN activity during rest and stepping

**Figure 2.**
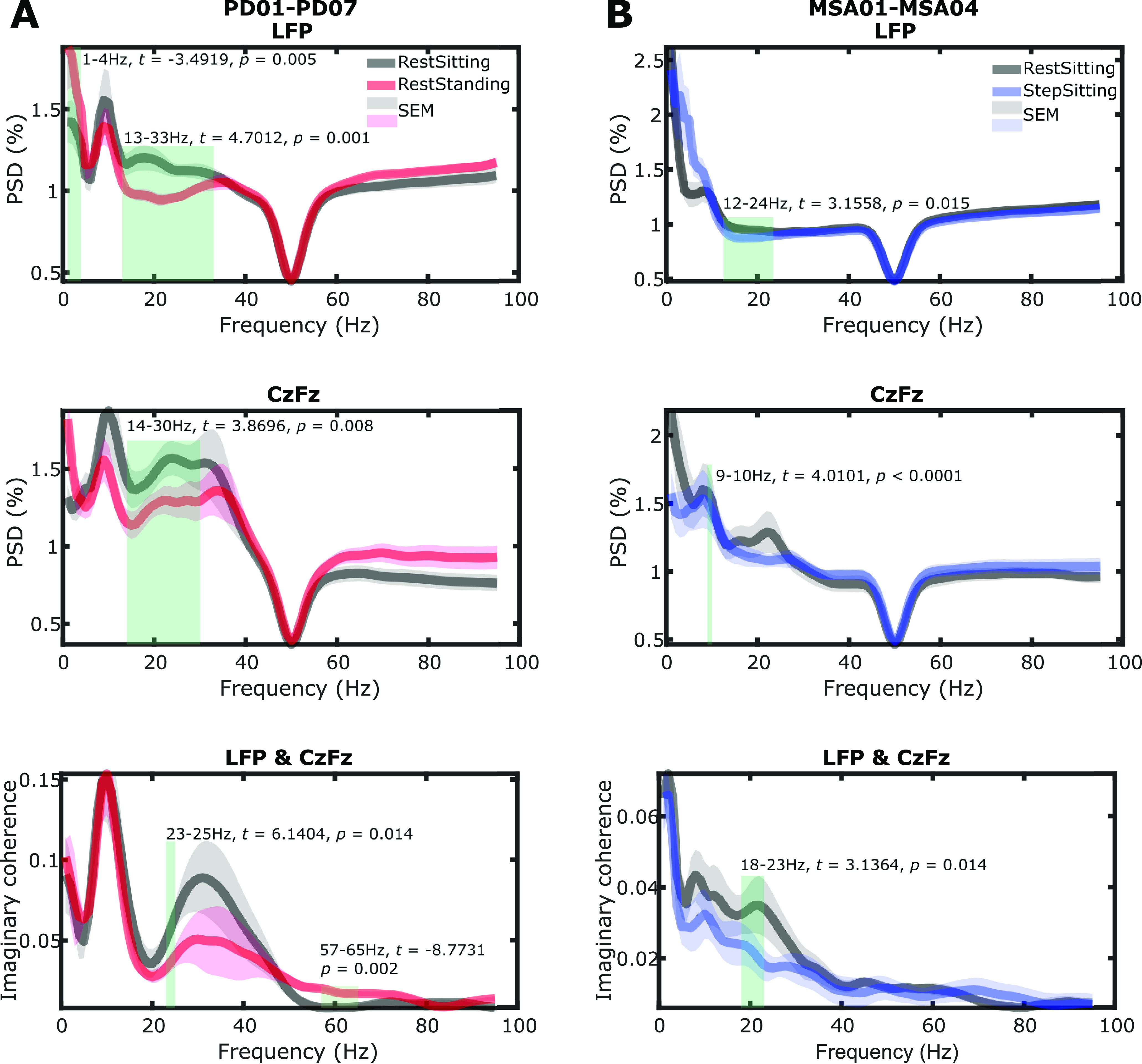
Power spectral density and coherence between LFP and EEG. ***A***, PSD of PPN activity (top), cortical EEG activity (middle), and imaginary coherence (bottom) between PPN and cortical EEG during rest while sitting (black) and rest while standing (red) averaged across seven PD patients (12 hemispheres). ***B***, PSD and imaginary coherence during rest while sitting (black) and stepping while sitting (blue) averaged across four MSA patients (eight hemispheres). Green rectangle indicates significant difference based on a cluster-based permutation procedure.

During standing or stepping compared with resting while sitting, the LFP activities in the PPN and PPN–cortical coherence were modulated in the beta band for both patient groups (Fig. 2). Peaks in the alpha frequency band were observed in the power spectra density in PPN LFPs recorded from PD patients during standing and sitting, and this was less obvious in the recordings from MSA patients. Figure 2*A* shows that for PD patients, the power in the broad beta band in both PPN LFPs and cortical EEGs was significantly reduced while standing compared with sitting. The imaginary coherence between PPN LFPs and cortical EEGs was significantly reduced in the beta band (23–25 Hz, *t* = 6.1404, *p* = 0.014) and increased in the gamma band (57–65 Hz: *t* = −8.7731, *p* = 0.002). In the MSA patients, the power in a narrow beta band in the PPN (12–24 Hz: *t* = 3.1558, *p* = 0.015) and the imaginary coherence in beta band between PPN LFPs and cortical EEGs (18–23 Hz: *t* = 3.1364, *p* = 0.014) were significantly reduced during stepping compared with rest while sitting (Fig. 2*B*).

### Modulation of PPN LFP activities within gait cycles

**Figure 3.**
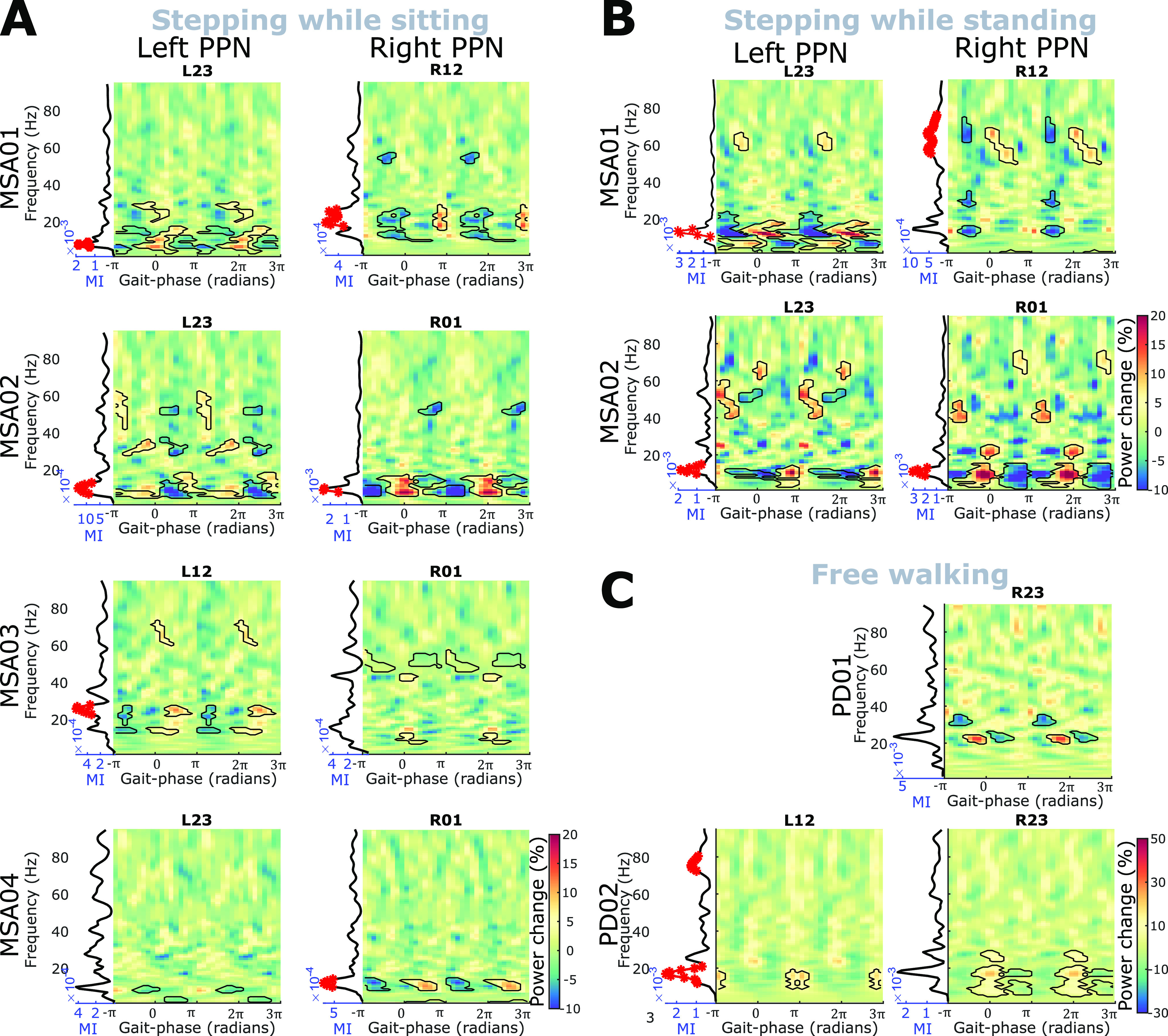
Power modulation during gait for the bipolar channel showing the strongest modulation effect in each tested PPN. ***A*–*C***, Similar modulation was observed during stepping while sitting (***A***), stepping while standing (***B***), and free walking (***C***). In each subplot, a modulogram (one bipolar channel from each hemisphere) showing the power in PPN LFPs modulated by the gait phase, with zero indicating the heel strike and –π (π) indicating the heal lift of the contralateral foot. The line plot to the left of the modulogram shows the corresponding modulation index. The black contours and red dots indicate significant modulation based on a cluster-based permutation procedure (*p* < 0.05).

The LFP activity–gait phase modulogram and the modulation index were quantified for each of the individual bipolar PPN LFPs based on the stepping/walking phase of the contralateral foot. During stepping while sitting (Fig. 3*A*), stepping while standing (Fig. 3*B*), or free walking (Fig. 3*C*), significant modulation was observed in all hemispheres from all patients, with the strongest modulation in the alpha and beta bands. The modulation pattern in the PPN LFPs recorded during stepping on spot while standing (Fig. 3*B*) was very similar to what was observed during stepping while sitting (Fig. 3*A*) for the same contacts in the same patient. Rhythmic modulation with gait cycle was more often observed in the alpha and beta frequency band, but can also be observed in the gamma frequency band in some contacts. Note that PD01 received only a unilaterally implanted electrode.

**Figure 4.**
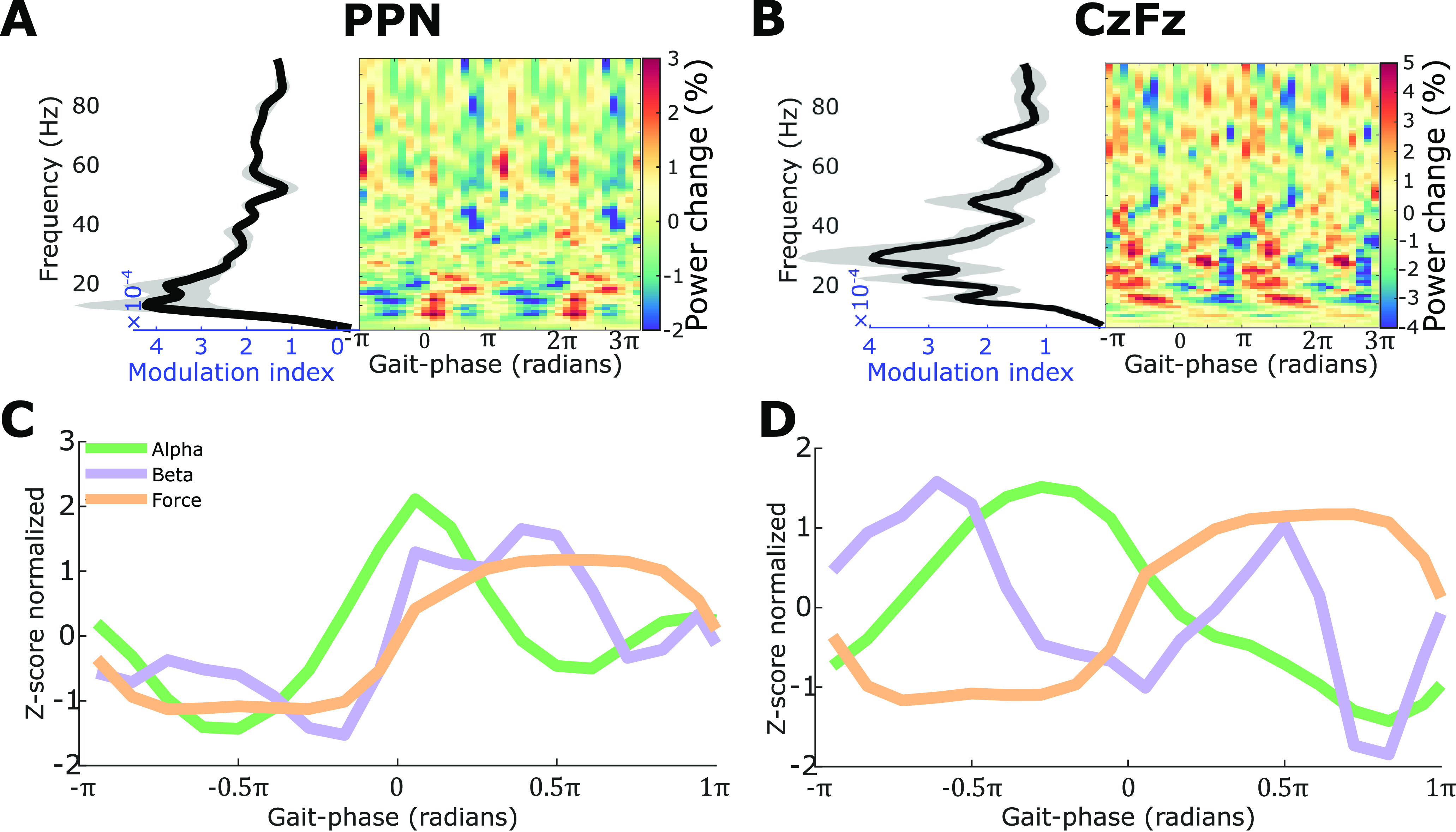
Averaged power modulation during stepping while sitting (*n* = 8 PPN of four MSA patients). ***A***, ***B***, Power was modulated in both PPN LFP (***A***) and cortical EEG (***B***). ***C***, ***D***, Averaged power in alpha (green) and beta (purple) frequency bands, and the averaged force (orange) relative to gait phase in PPN (***C***) and cortical EEG (***D***).

To compare the modulation between PPN and cortex, we averaged the time–frequency plots and the modulation indices across all LFP channels for each hemisphere, and then averaged across all hemispheres and compared it with the averaged result from EEG. As shown in Figure 4, the gait phase-related modulation index was highest in alpha and beta bands in both PPN and cortex (Fig. 4*A*,*B*). There was one peak in the power of alpha and beta band activities within one gait cycle in the PPN (Fig. 4*C*). However, two peaks in the beta band were observed in the cortical signals (Fig. 4*D*, purple line). Leg movements are represented in the medial part of the primary motor cortex (He et al., 1995), which makes it difficult to separate activities related to left and right leg movements with a sparse spatial coverage of EEG electrodes. Superposition of activity corresponding to the movements of the left and right leg may explain the two peaks in cortical activity.

### More regular stepping tends to be associated with stronger gait phase-related modulation in PPN LFPs

**Figure 5.**
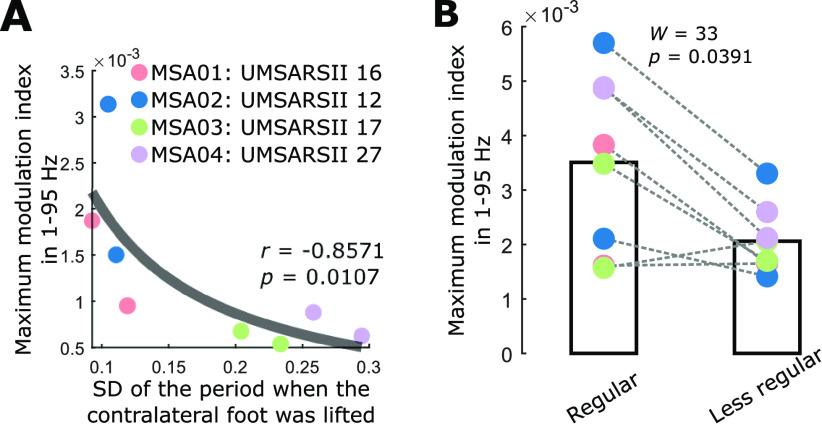
More regular stepping tends to be associated with stronger gait phase-related modulation in PPN LFPs. ***A***, Relationship between the maximum modulation index and the variability of the stepping while sitting. The *y*-axis indicates the maximum modulation index in a wide frequency band (1–95 Hz), and The *x*-axis indicates the SD of the period when the contralateral foot was lifted. Different colors indicate the points for different patients. The gray curve is a 1/*x* fitting of all data points. ***B***, The maximum modulation index during more regular stepping was significantly larger than during less regular stepping (Wilcoxon signed-rank test). Patients are color coded as in ***A***.

To investigate the impact of the stepping variability on the observed modulation in PPN LFPs, we first quantified the SD of the duration of the period when the contralateral foot was lifted (i.e., the periods when the filtered force was negative) across all stepping cycles for all patients with MSA and correlated them with the maximum modulation index in a wide frequency band (1–95 Hz). We computed the correlations with the maximum modulation index rather than the mean of individual bands because significant modulation appeared in relatively narrow frequency bands (Fig. 3). As shown in Figure 5*A*, the two patients showing a stronger modulation effect (MSA01 and MSA02) had a smaller stepping variability as well as smaller gait impairment scores, indicating less severe motor impairment based on the unified MSA rating scores [motor examination scale (part II) of unified MSA rating scale (UMSARS-II); Table 1]. Across the eight hemispheres, we found a negative correlation between the stepping variability and the maximum modulation index (*r* = −0.8571, *p* = 0.0107, Spearman's correlation). Then, we split all stepping cycles of each leg into a more regular group and a less regular group with 25% cycles (49.25 ± 1.66, mean ± SEM) in each group for each hemisphere and compared the maximum modulation index between these two conditions. The more/less regular group was split based on the difference between the duration of each stepping cycle and the average duration across all cycles, with smaller/larger values representing the more/less regular group (mean ± SD: regular group, 0.0259 ± 0.0036 s; less regular group, 0.3080 ± 0.0507 s; *W* = 0, *p* = 0.0078, Wilcoxon signed-rank test). As shown in Figure 5*B*, the maximum modulation index was significantly higher during more regular stepping (*W* = 33, *p* = 0.0391, Wilcoxon signed rank test). These results suggest that more regular stepping tended to induce stronger modulation in PPN.

### Clusters of LFP states indicating different gait phases were identified using HMM

**Figure 6.**
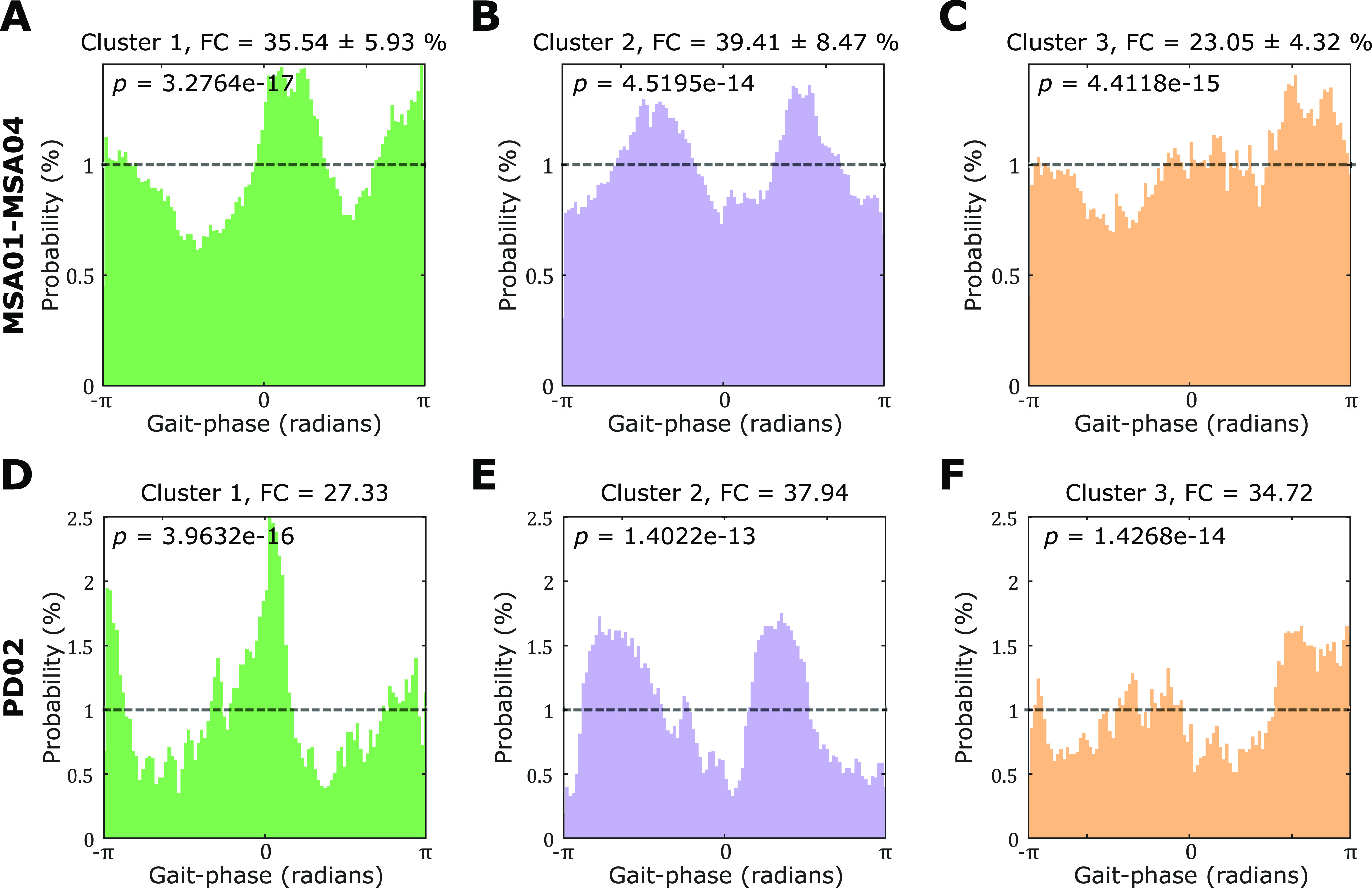
Clusters with different occurrence rates at different gait phases were identified using unsupervised machine learning algorithms (HMM and K-medoids clustering). ***A***, Cluster 1 showed states with higher probabilities around phase –π/π (when the foot was lifted) and phase 0 (at the time of the heel strike) during stepping while sitting. ***B***, Cluster 2 showed higher probabilities around phase –π/2 (when the foot was highest in the air) and π/2 (in the middle of the stance period) during stepping while sitting. ***C***, Cluster 3 did not show specific preferred gait phases during stepping while sitting. The results in ***A***–***C*** were averaged across all MSA patients. ***D***–***F***, Very similar results were obtained for one PD patient during free walking. Note that each histogram consisted of 100 phase bins. The black dashed line indicates the uniform probability (i.e., 1%) of each phase bin, which would be expected if no modulation effect was present. Kolmogorov–Smirnov tests revealed that the observed distributions were all significantly different compared with a uniform distribution.

The unsupervised machine learning algorithms (HMM and K-medoids clustering) identified clusters of PPN LFP states with the highest occurrence at opposite points of the stepping/walking phases (Fig. 6). Specifically, states in cluster 1 were more likely to be detected around phases –π and π (when the foot is lifted), and around phase 0 (at the time of the heel strike; Fig. 6*A*). The occurrence of cluster 2 states was most probable at around –π/2 (when the foot was highest in the air) and π/2 (in the middle of the stance period; Fig. 6*B*). There was no significant difference in terms of the state time fractional occupancies indicating how much time each subject spends in each state (FCs) between these two clusters (cluster 1, FC = 35.16 ± 5.13%; cluster 2, 35.86 ± 11.75%). A similar pattern was detected in the PD patient (PD02; Fig. 6*D*,*E*). We did not include PD01 here because the number of recorded walking cycles (25) was too small to provide reliable state estimates.

### Alpha and beta modulation were clustered in the PPN

**Figure 7.**
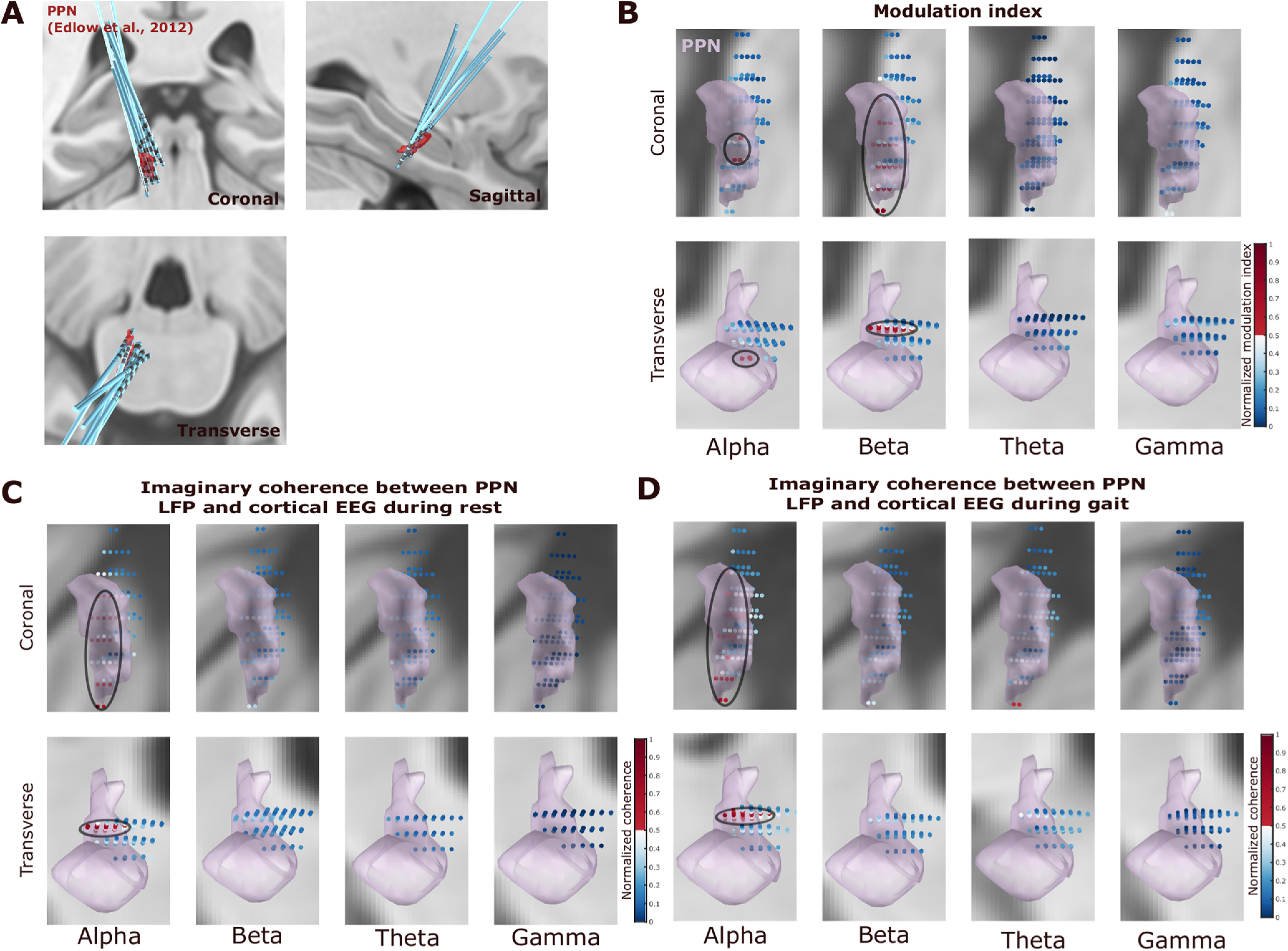
***A–C***, Electrode localization (***A***) and the mapping of the modulation index (***B***) as well as the PPN–cortical coherence (***C***) in standard MNI space. ***A***, 3D reconstruction in coronal (top left), sagittal (top right), and transverse (bottom left) views of all the recorded DBS leads from 10 hemispheres (five patients: MSA, four patients; PD, one patient) using Lead-DBS software. ***B***, The bipolar contacts close to the PPN in the MNI space tend to show higher gait phase-related modulation index in alpha (8–12 Hz) and beta (13–30 Hz) frequency bands, and the modulation index in these two frequency bands were stronger compared with theta and gamma frequency bands. ***C***, The imaginary coherence between PPN LFP and cortical activities (measured at CzFz using EEG) averaged in the alpha frequency band was clustered close to PPN in the MNI space and was strongest compared with the beta, theta, and gamma frequency bands. ***D***, The same holds for the imaginary coherence between PPN LFP and cortex EEG during gait. Black ovals indicate clusters of larger values. The two rows show the results in two different views.

**Figure 8.**
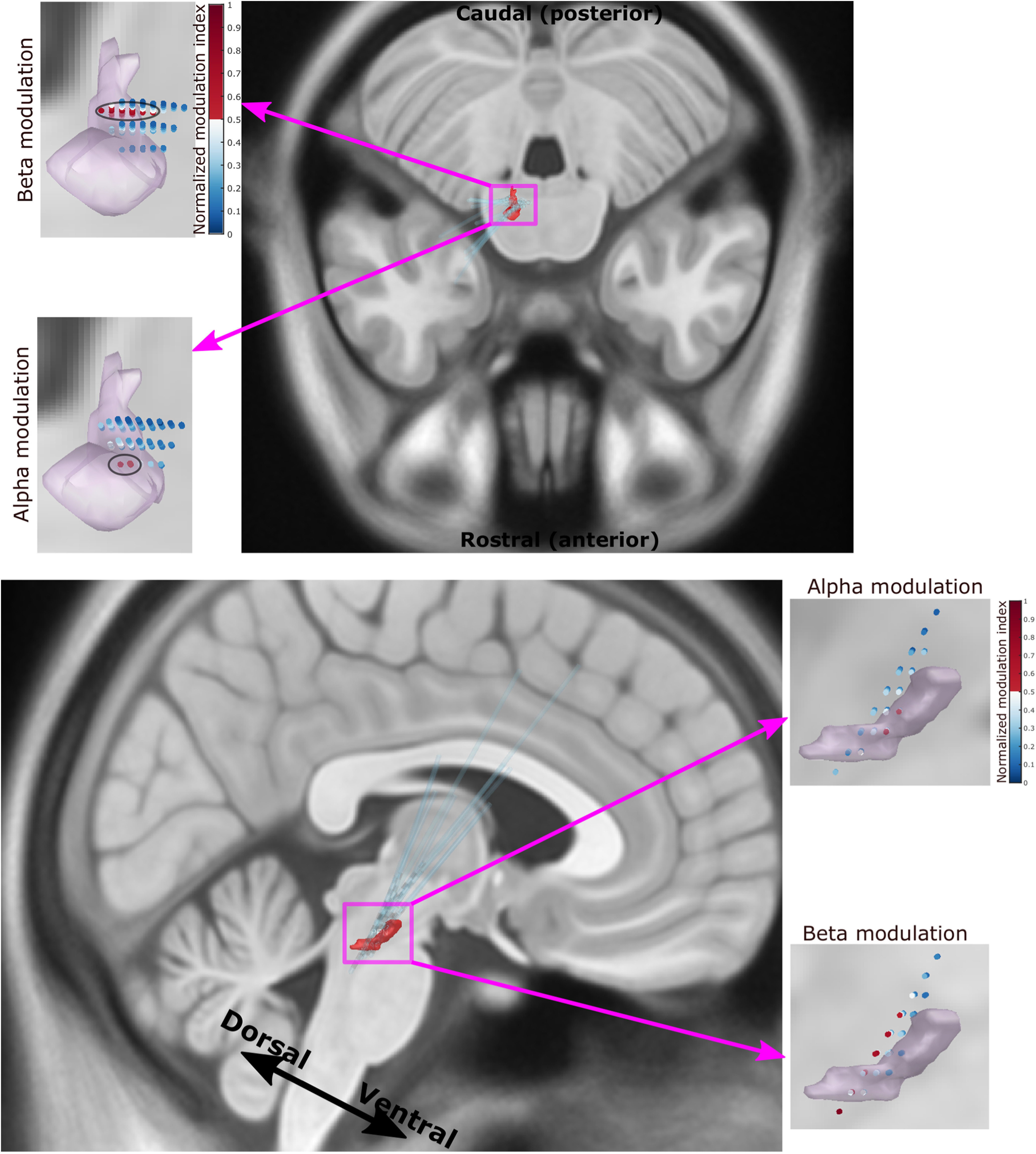
The strongest modulation effect in alpha band was more focused in the ventral–rostral portion of the PPN while the strongest modulation in beta band was a more widely spatial spread in the dorsal–caudal PPN.

Electrode location reconstruction using the LeadDBS software (Horn et al., 2019) confirmed that most electrodes were well placed in the PPN area with respect to the Harvard AAN Atlas (Edlow et al., 2012), as shown in Figure 7*A*. The gait-related power modulation was mainly observed in alpha and beta frequency bands but not in theta and gamma frequency bands (Fig. 7*B*). There was a wider spatial spread of gait phase-related modulation in the dorsal–caudal PPN plane in the beta band activities during stepping or walking; in comparison, the modulation in alpha band activities was more focused in the ventral–rostral plane of the PPN (Fig. 8). In addition, the imaginary coherence between PPN LFPs and cortical EEGs during rest or gait mainly occurred in the alpha band, but not in the beta, theta, or gamma bands (Fig. 7*C*,*D*).

## Discussion

We found that alpha and beta oscillations in the PPN LFP are modulated relative to the contralateral foot gait phase within gait cycles in patients with MSA and PD during stepping movements, no matter whether they were seated or standing, or during free walking. Gait phase-related modulation increased with more regular stepping movements, was specific to alpha and beta frequency bands, and clustered around the PPN area defined by the Harvard AAN Atlas. Consistent findings from two patient groups in different tasks suggest that the gait phase-related modulation in the PPN LFPs can be related to normal stepping movements. These results are similar to those we have observed in STN (Fischer et al., 2018). The gait phase-related modulation in the STN was strongest in the high beta band between 20 and 35 Hz (Fischer et al., 2018), whereas gait-phase modulated PPN activity was specific to the alpha and low beta range. The modulation reflects neural activity and not movement-related artifacts as artifacts are minimal during stepping while sitting (Fischer et al., 2018), and the modulation pattern was specific to limited frequency bands. The results of this study are also in line with reports that alpha oscillations in the PPN are associated with gait in PD patients (Thevathasan et al., 2011, 2012; Lau et al., 2015; Li and Zhang, 2015). However, previous studies focused on PPN power averaged across the whole walking period, and hypothesized that increased alpha activity in the PPN is important for normal gait, and that low-frequency PPN DBS supports or emulates this activity, consequently enhancing the allocation of attentional resources and improving gait performance (Thevathasan et al., 2018; Thevathasan and Moro, 2019). Here we provide novel evidence for gait phase-related modulation of PPN activities in the alpha and beta bands for regular stepping. Transiently increased activity in the beta frequency band in the cortical–subcortical motor network, including the STN, PPN, and cortex may help to maintain accurate and discrete representations of muscle synergies (Aumann and Prut, 2015), and allow binding task-related motor neurons into functional units to facilitate muscle coordination (Bourguignon et al., 2017; Reyes et al., 2017). Modulation of beta band activities by the phase of gait cycles may support different muscle synergies for dynamic versus static movement periods (Kenville et al., 2020a,b) that are alternating in gait. Previous studies suggest that the PPN integrates both sensory and motor information via connections to almost all other parts of the CNS (Benarroch, 2013; neurology). It has been shown that PPN neuronal activities can be modulated by sensory stimuli, imaginary gait, and passive lower limb movement, and PPN DBS can modulate the response of PPN activity to sensory stimuli (Grunwerg et al., 1992; Reese et al., 1995; Tattersall et al., 2014; Lau et al., 2015; Strumpf et al., 2016; Yousif et al., 2016). Thus, it is not clear from the current study whether the observed PPN LFP modulation represents the efferent motor command or is an outcome of sensory feedback during stepping or free walking.

### Location

The boundaries of the PPN are still indistinct, and there is inconsistency in the location of the PPN in different atlases (Thevathasan and Moro, 2019). In this study, we used the PPN atlas defined in Lead DBS according to a study by Edlow et al. (2012) to reconstruct the trajectories of the DBS electrodes. With this atlas, we showed that the gait phase-related modulation was more focused in the ventral–rostral plane of the PPN in the alpha band and exhibited a wider spatial spread in the dorsal–caudal PPN plane in the beta band (Figs. 7*B*, 8). It should be noted that the definition of the PPN borders in standard MNI space in other atlases may be slightly different compared with the one we were using (Snijders et al., 2016; Alho et al., 2017; Ilinsky et al., 2018). In this study, we chose the atlas occupying the largest volume in the standard space. Nevertheless, since precise localization of PPN is difficult, the structures defined in all these atlases should be considered presumptive, with the atlas we chose being the most inclusive one.

### Detecting the gait phase using PPN LFPs

We applied unsupervised HMMs and K-medoids on the raw PPN LFPs and identified two clusters with complementary preferred gait phases (Fig. 6). This suggests that gait-related information in the PPN LFPs can be identified by HMM, which further demonstrated that the PPN activities were modulated during gait. Although the modulation was most consistently observed in alpha and beta bands across patients, significant modulation was also observed at other frequency bands such as theta and gamma in some contacts and some patients (Fig. 3). Applying HMM on the raw time series from all LFP measurements theoretically has the benefit of incorporating all features in the signal, including activities in different frequency bands and cross-frequency coupling between activities from different recording channels. In addition, the results of unsupervised HMM were directly achieved based on the raw time series without using the information for the gait phase in the models, suggesting that it is possible to detect gait phase from PPN LFPs.

### Potential application in closed loop DBS for gait control

Recently, in PD patients, patterns of beta modulation in the left and right STNs were shown to peak at alternating points of the stepping cycle (Fischer et al., 2018). Furthermore, an alternating DBS pattern, consisting of rhythmic intermittent reductions in stimulation intensity with a fixed offset between the right and left STN, was shown to significantly manipulate the step timing (Fischer et al., 2020). These results have raised the possibility that alternating stimulation in the STN may be a promising DBS pattern for gait control in PD. In this study, we showed that alpha and beta oscillations in the PPN were modulated relative to the gait phase of the contralateral foot in both patients with MSA and PD.

Notably, the STN modulation found in our previous study was strongest in the high beta band (Fischer et al., 2018), between 20 and 35 Hz, while PPN activity also was modulated in the alpha range and showed a distinct alpha power peak. STN beta activity tends to be suppressed by STN DBS (Kühn et al., 2008), but it is unclear to what extent PPN alpha/beta activity is modulated by PPN DBS, particularly considering that stimulation frequencies can vary between 20 and 80 Hz (Thevathasan et al., 2018).

Recently, another independent study reported that, compared with continuous DBS, gait phase-triggered responsive DBS targeting STN or globus pallidus internus leads to significant improvement in gait in only 3 of 12 participants (Louie et al., 2021). However, the stimulation was switched ON for only 12% of each cycle in the fixed gait phase, which might not be enough to enhance the alternating pattern of the brain activities during stepping. In addition, we also showed that the gait phase, with reduced/enhanced activities, was variable across participants and across different contacts within one participant, suggesting that the optimal phase and contact for stimulation should be selected for each participant individually.

The PPN has specific relevance to locomotion, as it is considered a key component of the MLR—an area where electrical stimulation in decerebrated animals can induce locomotor-like activity (Garcia-Rill et al., 1987). It has widespread reciprocal connectivity with many structures, including basal ganglia nuclei, thalamus, nuclei of the pontine and medullary reticular formations, deep cerebellar nuclei, and the spinal cord (Pienaar et al., 2017). Thus, the PPN may be a more powerful target for DBS than the STN in improving gait. Further studies as to how PPN DBS affects activities in the PPN and motor network, how the effects of stimulation change with the stimulation frequency (Thevathasan et al., 2018), and how alternating PPN DBS might affect the observed alternating PPN modulation as well as the stepping performance are warranted.

### Limitations

The first limitation of this study other than the uncertainty in PPN spatial limits considered above is our small sample size. We analyzed data from two PD patients and four MSA patients to quantify gait phase-related modulation in the PPN LFP. PPN DBS is still an emerging experimental treatment for patients with gait problems, and the number of patients operated on is small (Thevathasan et al., 2018). The MSA patients were involved in a recently registered clinical trial to investigate the clinical effect of PPN DBS on gait control. Because of the strict inclusion criteria of the trial and the impact of the pandemic, we were only able to record four patients in this cohort. The PD patients were originally recruited for some other studies (Thevathasan et al., 2011, 2012), and thus we managed to perform gait phase-related analysis in only two of seven PD patients. However, the results were consistent within this small sample size, and modulation was consistently observed across stepping movements, made while seated, standing, or free walking. The second limitation is that this study is only correlational. It is impossible to distinguish whether the modulation we observed reflects motor output or sensory feedback about the movements. Further studies are required to test the causal role of rhythmic modulation of PPN activity and its potential clinical benefits. For instance, we could investigate the PPN activities during passive leg movements to assess this or to test alternating PPN DBS patterns and evaluate their impact on gait control, similar to what was previously investigated with alternating STN DBS (Fischer et al., 2020).

### Summary

This study provides new evidence that PPN activities in the alpha and beta bands are modulated by gait phase within gait cycles, with the modulation increased during more regular stepping movements. Our observations raise the possibility that modulating PPN DBS relative to the gait cycle mimicking the modulation pattern we observed during stepping could potentially facilitate the observed rhythmic pattern better than continuous stimulation, although whether this could lead to further improvement in gait performance remains to be established in future studies.
